# Electrospun Zein/Polyoxyethylene Core-Sheath Ultrathin Fibers and Their Antibacterial Food Packaging Applications

**DOI:** 10.3390/biom12081110

**Published:** 2022-08-12

**Authors:** Wenlai Jiang, Ping Zhao, Wenliang Song, Menglong Wang, Deng-Guang Yu

**Affiliations:** 1 School of Materials & Chemistry, University of Shanghai for Science & Technology, Shanghai 200093, China; 2 Shanghai Engineering Technology Research Center for High-Performance Medical Device Materials, Shanghai 200093, China

**Keywords:** polyethylene oxide, zein, electrospinning, antibacterial, food packaging

## Abstract

The purpose of this work is to develop a novel ultrathin fibrous membrane with a core-sheath structure as antibacterial food packaging film. Coaxial electrospinning was exploited to create the core-sheath structure, by which the delivery regulation of the active substance was achieved. Resveratrol (RE) and silver nanoparticles (AgNPs) were loaded into electrospun zein/polyethylene oxide ultrathin fibers to ensure a synergistic antibacterial performance. Under the assessments of a scanning electron microscope and transmission electron microscope, the ultrathin fiber was demonstrated to have a fine linear morphology, smooth surface and obvious core-sheath structure. X-ray diffraction and Fourier transform infrared analyses showed that RE and AgNPs coexisted in the ultrathin fibers and had good compatibility with the polymeric matrices. The water contact angle experiments were conducted to evaluate the hydrophilicity and hygroscopicity of the fibers. In vitro dissolution tests revealed that RE was released in a sustained manner. In the antibacterial experiments against *Staphylococcus aureus* and *Escherichia coli*, the diameters of the inhibition zone of the fiber were 8.89 ± 0.09 mm and 7.26 ± 0.10 mm, respectively. Finally, cherry tomatoes were selected as the packaging object and packed with fiber films. In a practical application, the fiber films effectively reduced the bacteria and decreased the quality loss of cherry tomatoes, thereby prolonging the fresh-keeping period of cherry tomatoes to 12 days. Following the protocols reported here, many new food packaging films can be similarly developed in the future.

## 1. Introduction

The requirement for food quality and safety is always one of the biggest concerns for consumers. To ensure fresh, fragrant and nutritious food production, bio-based food packaging materials have been widely studied, enabling a defense against the volatile environment in food production [1,2,3]. Resveratrol, a non-flavonoid polyphenol organic compound, is a natural antitoxin that can be extracted from plants, mainly including Polygonum Cuspidate and grapes [4]. In addition, it has anti-inflammatory, anti-cancer and cardiovascular-protective effects on the human body, as well as antibacterial and antioxidant properties in organisms [5,6,7]. Therefore, there is a great arousal of interest among researchers to apply resveratrol to food packaging materials, which is advantageous for both health needs and active packaging requirements.

Electrospinning is a direct and easy-to-implement, top-down nanotechnology powered by electrostatic energy and involves fluid dynamics, which has been popular over the past decade [8,9,10,11,12]. It is not only a micro/nanofiber forming technology, but also can be used as a micro/nanocomponent encapsulation technology. Once a biocompound such as resveratrol is added into an electrospun precursor solution, it can be highly evenly dispersed in the fiber in the form of solid dispersion through electrospinning preparation [13,14]. Considering the conditions of selecting appropriate hydrophilic or hydrophobic polymers and specific humidity in the packaging environment, we can artificially control the release rate of resveratrol to achieve the expected packaging effect of prolonging the shelf life of food. Meanwhile, factors such as resveratrol being insoluble in water and unstable in light can be solved through electrospinning [15,16,17].

Electrospinning technology has developed into multi-fluid electrospinning technology that is applied in many fields, such as drug delivery [18,19,20,21,22,23,24] that is coaxial [25,26,27], modified coaxial [28,29], parallel [30,31], triaxial [32,33] and so on. The drug release rate can be controlled by adjusting the structure of nanofibers through multi-fluid electrospinning, which possesses certain advantages in the preparation process [34,35,36]. In the active food packaging field, this idea is also applicable. We can regulate the structure to control the release rate of active substances to meet the requirements of various kinds of food packaging [37]. Han et al. [38] prepared the fiber film for packaging aquatic products by coaxial electrospinning, and used the core-sheath structure to mask the pungent smell of the antibacterial component cinnamaldehyde. Patiño et al. [39] used coaxial electrospinning technology to slow down the release rate of encapsulated substances to the simulant by using a core–shell structure for the food packaging system.

Food-grade polymer is an edible carrier matrix which has been widely studied in the field of food packaging [40,41,42,43]. It is mostly extracted from natural plants that include polysaccharides and proteins, which are compatible with the requirements of food packaging materials [44,45]. Zein is a hydrophobic macromolecular protein. Compared with other proteins, it is easier to extract and is economical [46]. In addition, it also has good biocompatibility. However, its brittleness is high and its electrospinnability is slightly insufficient. Therefore, it is commonly considered necessary to improve the properties of zein by compounding it with synthetic polymers possessing high mechanical strength [47]. PEO is a biodegradable polymer with strong electrospinnability and good mechanical properties [48]. The blend electrospinning of zein-PEO has not been studied yet. Therefore, in this paper, a zein-PEO core-sheath nanofiber fabricated by coaxial electrospinning was studied as a composite substrate.

The method of filling nanoparticles into packaging materials to prolong food shelf life with an antibacterial effect is receiving more and more attention [49], and silver nanoparticles are one of these being studied [50]. Nanosilver is an effective antifungal and antibacterial agent, which can inhibit the synthesis and metabolic activities of cellular proteins, nucleic acids and other substances to quickly kill a single microorganism [51]. According to research, nanosilver is nontoxic under the condition of controlling a certain dose. Its application in food packaging is gradually developing [52,53]. Polyethylene films loaded with nanosilver particles with different concentrations were prepared in [54], which proved that it can effectively reduce the number of microorganisms in packaging. It was reported that cellulose acetate/nanosilver composite films have strong antibacterial activity and nontoxicity.

Based on previous research, this study aims to prepare core-sheath electrospun PEO-zein ultrathin fibers loaded with resveratrol and silver nanoparticles simultaneously in order to achieve an efficient antibacterial packaging effect. The composition and morphology of the fiber were characterized, and the release curve of resveratrol in the fiber was studied. In a practical application, cherry tomatoes were selected as the target packaging food.

## 2. Materials and Methods

### 2.1. Materials

Zein (from corn) was purchased from Tokyo Chemical Industry Co., Ltd. (Tokyo, Japan). PEO (Mw = 1,000,000) was purchased from Sinopharm Chemical Reagent Co., Ltd. (Shanghai, China). Resveratrol (>98%) was supplied from Xi’an Tianxingjian Natural Bio-products Co., Ltd. (Shaanxi, China). Ethanol was provided from Shanghai Sinopharm Co., Ltd. (Shanghai, China). All chemicals were of analytical grade, and water was distilled doubly through a purification system. AgNPs (96.5 wt %) were bought from Jiangsu Xianfeng Nano Material Technology Co., Ltd. (Jiangsu, China). The test bacteria of *Staphylococcus aureus* (*S. aureus*, CMCC26003) and *Escherichia coli* (*E. coli*, CMCC44102) were provided by Shanghai Sixth People’s Hospital. Fresh cherry tomatoes were purchased from the local market in Shanghai near our university.

### 2.2. Electrospinning Preparation Process

The homemade electrospinning system was used to fabricate fibers, which was composed of 4 parts: A high-voltage (60 kV/2 mA) power supply (ZGF2000, Shanghai Sute Corp., Shanghai, China), two syringe pumps (KDS100 and KDS200, Cole-Parmer, Lake County, IL, USA), a homemade concentric spinneret and a piece of aluminum foil wrapped on the cardboard as a collector [55,56,57,58].

The two working fluids were arranged as the core solution and sheath solution to fabricate fibers through the homemade spinneret. In the core solution, 3% *w*/*v* PEO was dissolved in an 80% *v*/*v* ethanol aqueous solution, and 10% *w*/*w* resveratrol was added into the solution and stirred on a magnetic stirrer overnight. In the sheath suspension, the purchased nanosilver was placed in a mortar for grinding to facilitate a better dispersion. An amount of 0.1% *w*/*w* nanosilver was suspended into the 80% *v*/*v* ethanol aqueous solution, with 28% *w*/*v* zein and 5% *w*/*w* resveratrol. The coblended liquid was stirred on the magnetic stirrer overnight after 2 h of ultrasound. Before adding the solution into the syringe, it was ensured that the substances in the solution were uniformly mixed and free of bubbles. The experiment was carried out at 24 ± 2 °C. The control group was prepared by adjusting the content of resveratrol in the core or sheath solution. The detailed parameters are listed in Table 1.

With a series of pre-experiments, including uniaxial and coaxial electrospinning, the electrospinning process parameters were gradually optimized to prepare the zein-PEO fiber. The voltage was set at 7.0 kV; the collection distance was 23 ± 2 cm; the core fluid flow rate was 0.6 mL/h; the sheath fluid flow rate was 0.3 mL/h; the ambient temperature was 24 ± 2 °C; the relative humidity was 60 ± 5%.

### 2.3. Morphology and Structure

All the electrospun fiber membranes were measured through the Quanta FEG 450 scanning electron microscope (SEM, FEI Co., Ltd., Hillsboro, OR, USA). Before the SEM characterization, the samples were first sprayed on a thin layer of gold using vacuum evaporation equipment. The average fiber diameter distribution was measured through ImageJ software (National Institutes of Health, Bethesda, Rockville, MD, USA) by selecting 100 fibers in the SEM photos.

### 2.4. Physical Status and Compatibility

Attenuated total reflectance Fourier transform infrared spectroscopy (ATR-FTIR, Spectrum 100, PerkinElmer, Waltham, MA, USA) was used to analyze the internal groups of electrospun fibers or raw materials. The scanning range was set from 450 to 4000 cm^−1^, with 8 scanning times.

An X-ray diffractometer (XRD, D/Max-BR, Shanghai Long Optical Instrument Co., Ltd., Shanghai, China) was used to analyze the crystallinity, elements or physical status of the prepared ultrathin fibers and raw materials. The incident angle 2θ was set in the range of 5–60°, whereas the voltage was 40 kV and the current was 30 mA.

### 2.5. Water Contact Angle

The water contact angle (WAC) of the sample was measured by an interface tension measuring instrument (JC2000C1, Shanghai Zhongchen digital technology equipment Co., Ltd., Shanghai, China). The measurement angle ranged from 0° to 180° with the test error ± 0.5°.

### 2.6. Release Behavior

For the purpose of the assessment of the release behavior of RE from the nanofiber membrane in the packaging environment with a certain humidity, the SHZ-86 Water Bath Constant-Temperature Shaker (Jintan Shuibei Science Popularization Experimental Instrument Factory, Changzhou, China) was used to study the RE dissolution property in distilled water. The RE-loaded fiber membranes were cut into rectangles, weighed to 25 ± 0.5 mg and placed in conical flasks containing 125 mL of deionized water. The flasks were then put into the shaker and immersed to a certain water line. Another flask containing deionized water was placed in the shaker to spare. The temperature was set at 25 ± 0.5°, whereas the rotation rate was fixed at 50 rpm. After a certain time interval, 2 mL of solution was collected out of the flask to measure the RE component concentration. At the same time, 2 mL of deionized water was taken out of the standby flask and added into the test solution to keep the total volume of the test solution constant [59,60,61,62].

For the calculation of the RE (*R*%) concentration in the test solution, the calculation formula is as follows:

(1) $$R\%=\frac{X_{n}\times V_{0}+\sum_{i=1}^{n-1}X_{i}\times V}{Q_{0}}\times 100\%$$

where *X_n_* represents the RE concentration of the solution transferred from the flask for the *n*-th time, and *X_i_* represents the concentration of the solution transferred from the flask for the *i*-th time. *V* represents the volume of the solution transferred each time from the flask, and *V*_0_ represents the total volume of the solution in the flask. *Q*_0_ represents the theoretical total mass quantity of RE.

### 2.7. Antibacterial Activity

The antibacterial activity of the electrospun PEO/zein nanofiber membrane against *E. coli* and *S. aureus* was studied by the agar diffusion method. All experimental instruments were sterilized first at a high temperature to achieve a sterile experimental environment. The nutrient medium was fixed on a Petri dish, and then a 100 μL bacterial suspension within the concentration of 1 × 10^7^ CFU/mL was poured onto the agar plate. Furthermore, 6 mm-diameter discs were punched with a hole punch from the fiber membrane, which ensured that the mass of the discs was kept the same. A group of 3 discs was placed on an agar dish and the plates were cultured for 24 h at 37°. The inhibition zone diameter of the plate was observed and recorded, through which the antibacterial activity of the nanofiber membrane could be analyzed qualitatively [63].

### 2.8. Application to Cherry Tomato Packaging

After the purchasing of fresh cherry tomatoes from the local market, which were weighed to be about 10 g, they were promptly packaged with PEO/zein nanofiber films. The nanofiber films were cut to be 10 cm × 10 cm. The packaging process lasted for 13 days under the condition of 25 ± 1 °C and 60 ± 1% humidity. The cherry tomatoes were assigned into four groups, with three parallel cherry tomatoes in each group. The first group of tomatoes was the control group without packaging that was left in the open air, whereas the second group was packed with F4, the third group was packed with F5 and the fourth group was packed with F6. At each preset time point, the cherry tomatoes were weighed. On the last day of the packaging experiment, each cherry tomato was put into the sterile normal saline and shaken for 1 h. As for the collected sample solution, the gradient dilution method and plate counting method were used to calculate the number of microorganisms on the surface of cherry tomatoes, with the counting unit set as lg CFU/g.

### 2.9. Statistical Analysis

The statistical analysis of the result data was conducted by Microsoft Windows Excel 2019 and OriginLab’s Origin 2021. Data were expressed as means ± standard deviation (SD). All experiments were conducted in triplicate.

## 3. Results and Discussion

### 3.1. Coaxial Electrospinning Process

**Figure 1:**
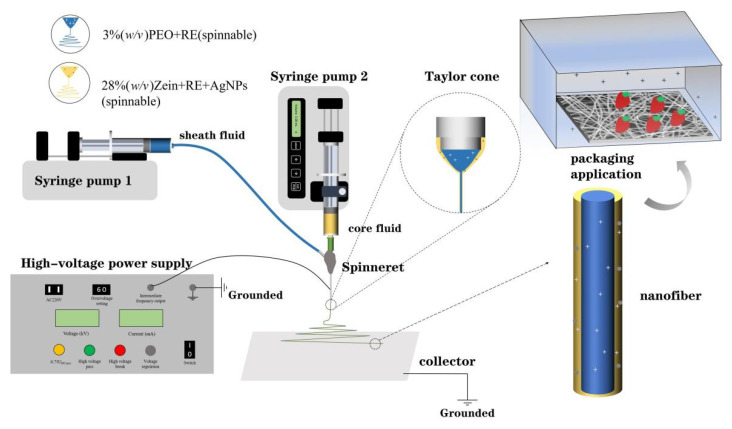
Schematic diagram of the coaxial electrospinning system and its application.

**Figure 2:**
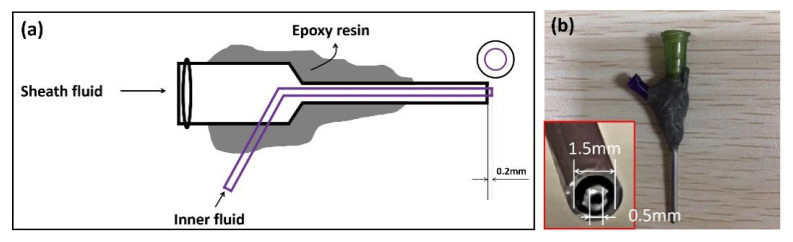
Structure of homemade coaxial spinneret: (**a**) diagram of designed detail structure of the coaxial spinneret; (**b**) coaxial spinneret and the head of the spinneret (bottom-left insert).

As illustrated in Figure 1, the core-sheath ultrathin fibers were prepared by the coaxial electrospinning process and were explored in the use for the preservation of cherry tomatoes. The coaxial electrospinning device consists of four parts: a power supply device for generating high-voltage static electricity, two syringe pumps for controlling the flow rate of the solution, a self-made coaxial spinneret with a separable solution and an aluminum foil collector for collecting fibers. Among them, the structure of the spinneret played a significant role in the whole electrospinning experiment process [64,65,66]. In the diagram illustrated in Figure 2, it can be seen how the coaxial spinneret was mainly composed of two metal capillaries. On the basis of the original uniaxial capillary with a diameter of 1.5 mm, an inner capillary with a diameter of 0.5 mm was inserted and encapsulated with epoxy resin to ensure that the relative position between the inner capillary and the outer capillary was fixed and that its cross-sectional center coincided (Figure 2b). Meanwhile, the existence of epoxy resin also ensured that the solution would not leak when it was pushed out of the spinneret at a certain flow rate. As shown in Figure 2a, the inner capillary overstepped 0.2 mm more than the outer capillary, which was designed to make the sheath solution form a ring-fluid structure more easily and to surround the inner solution more stably to further form a core-sheath-layered fluid. After solidification, the uniform core-sheath structure of ultrathin fibers could finally be obtained.

In the whole electrospinning process, the positive and negative poles of the high-voltage power supply were connected to the spinneret capillary and aluminum foil, respectively, which led to the formation of a high-voltage electrostatic field between the spinneret and aluminum foil [67,68,69]. As shown in Figure 1, the purpose of grounding was to introduce the charge on the aluminum foil into the ground and ensure a safer experimental environment. Driven by the syringe pump, the droplet of polymer solution was dropped down to the tip of spinneret at a certain flow rate, and was stretched into a Taylor cone under the effect of the high-voltage electrostatic force emitting a micro/nanojet. The jet would go through the whipping and bending process due to the electrostatic field force, reach the aluminum foil and transform into ultrathin fibers while rapid drying [70,71]. At the same time, during this process, the active substances uniformly dispersed in the solution would also maintain their morphology due to a high degree of solid dispersion that exists in ultrathin fibers [71,72]. In previous studies, it was concluded that only one fluid should be electrospinnable in coaxial electrospinning as the electrospinnability of the other fluid was not necessary, which was called modified coaxial electrospinning [73,74]. In this study, this theory was reflected. Despite the poor spinnability of zein in single electrospinning, the PEO fluid played a leading role in having strong spinnability to drive zein to form a continuous sheath structure of fibers around itself.

### 3.2. Morphology and Structure of Core-Sheath Ultrathin Fibers

**Figure 3:**
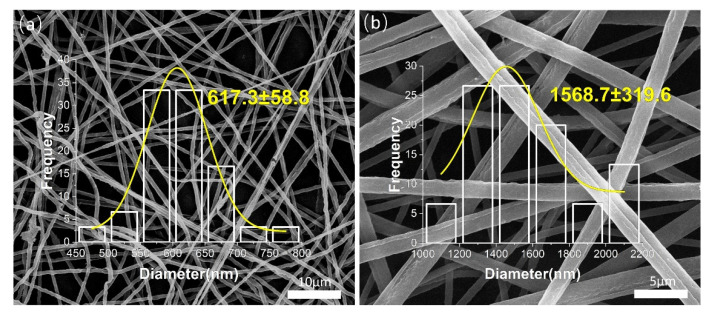
Characterization of F1 and F2 membrane by SEM: (**a**) F1—uniaxial fiber of PEO; (**b**) F2—coaxial fiber of PEO(core)/zein(sheath).

The SEM images of uniaxial ultrathin fibers and coaxial ultrathin fibers are shown in Figure 3. Figure 3a refers to PEO single-fluid electrospinning fibers, and Figure 3b refers to PEO/zein coaxial fibers, which were not loaded with active substances. The thickness inequality was found in uniaxial PEO fibers and the surfaces of the fibers were not smooth; thus, it could be inferred that the single-fluid electrospinning process of PEO was unstable. By contrast, the diameter distribution of the coaxial electrospinning fibers was uniform, and the diameters of fibers were larger than those fabricated by single-fluid electrospinning. This could be mainly due to the structural characteristics of the spinneret. The capillary diameter of the single-fluid spinneret was 0.8 mm, which was about half of the diameter of the outer capillary from the coaxial spinneret. Therefore, the Taylor cone formed by the solution at the tip of the coaxial spinneret was larger and the jet was thicker, and thus, the solidified fiber was also thicker [75,76]. The surface of the coaxial electrospun fiber was obtained wrinkled, which could be conducive to absorbing water when packaging food due to the increase in fiber surface area. In addition, it was beneficial for the release of active substances. The phenomenon of wrinkles might be caused by the volume shrinkage of zein during the rapid volatilization of the ethanol–water solvent.

**Figure 4:**
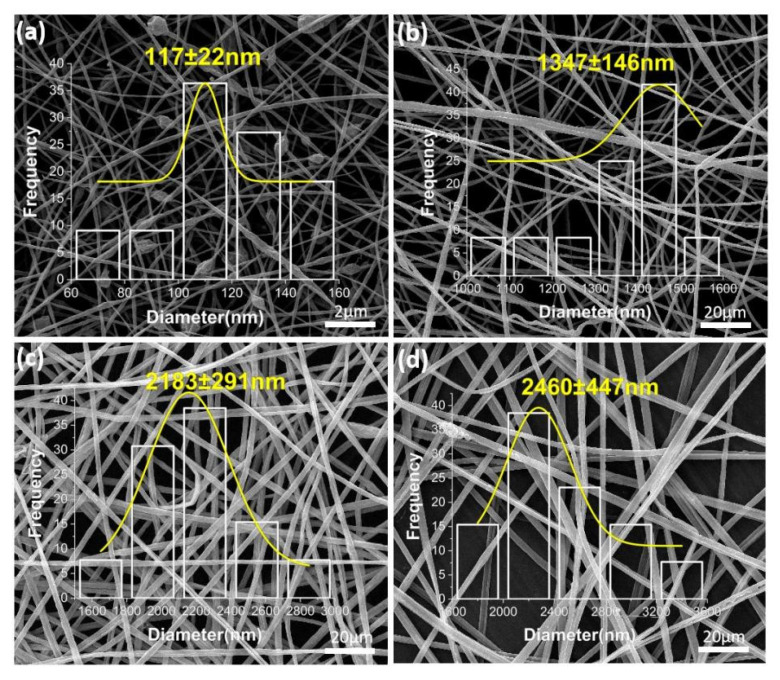
SEM images of the active-substance-loaded electrospun ultrathin fibers: (**a**) F3—zein-AgNP uniaxial nanofiber; (**b**) F4—PEO(core)/zein-AgNPs(sheath) coaxial nanofiber; (**c**) F5—PEO-RE(core)/zein-AgNP(sheath) coaxial nanofiber; (**d**) F6—PEO-RE(core)/zein-AgNP-RE(sheath) coaxial nanofiber.

As shown in Figure 4, on the basis of adding nano-silver, resveratrol was successively added to the core layer and sheath layer of the coaxial electrospinning fiber; Figure 4a–d correspond to F3–F6, respectively. As shown in Figure 4a, the zein fiber exhibited a bead-on-string structure, which was caused by the low viscosity of the zein solution and the electric field force possibly not being enough to stretch it into continuous fibers [77]. After adding PEO as the core layer, the dominant PEO fluid drove the sheath fluid of the zein layer under a strong fiber forming ability; therefore, zein could continuously surround the core layer. In addition, the fiber diameter was larger than that of the uniaxial fiber, as shown in Figure 4b. After a sufficient amount of RE being added to the core layer, as shown in Figure 4c, the diameter of fibers was about 2183 ± 291 nm. Afterwards, adding a small amount of RE to the sheath, as shown in Figure 4d, turned the diameter of the fiber to be about 2460 ± 447 nm. Furthermore, the fiber morphology became smoother, the diameter distribution was more uniform and the fiber diameter increased further. Part of the agglomerated nanosilver could be observed from the fiber surface, indicating an adequate load of nanosilver. It could be analyzed that there were chemical bonds between RE molecules and PEO and zein polymers. Enough RE molecules were interspersed in the polymer, which further promoted the stability of the polymer chain. Therefore, macroscopically, the fiber expanded in volume and showed a more stable and uniform morphology of fiber.

**Figure 5:**
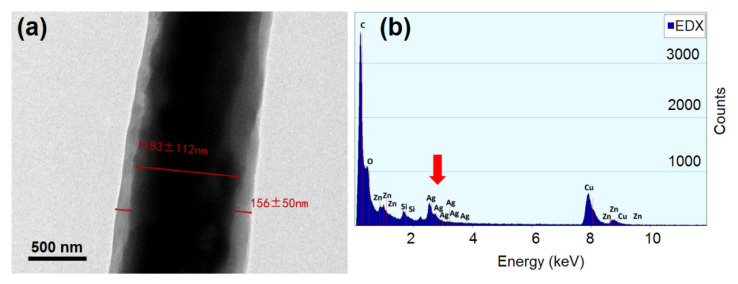
Internal structure and element analysis of the coaxial fiber: (**a**) TEM image of the coaxial nanofiber structure; (**b**) EDX energy spectrum of the coaxial fiber.

In order to further explore the internal structure of the fiber, TEM was used to analyze the fiber, with the F4 fiber selected as the sample. As shown in Figure 5a, the fiber owned an obvious core-sheath structure. The diameter of the core layer was about 1183 ± 112 nm and the sheath thickness was about 156 ± 50 nm. Zein turned out to be a nanocoating that wrapped around the core PEO fiber. To detect the local elements of the functional fiber, EDX analysis of the fiber was conducted. As Figure 5b shows, a silver element could be detected, indicating the successful loading of silver nanoparticles. The peak of the silver element was not tall enough compared to the carbon element, expressing the low content of AgNPs as experimentally designed. All in all, it was proven that the functional coaxial nanofiber was prepared successfully.

### 3.3. Physical State and Compatibility of Core-Sheath Ultrathin Fibers

**Figure 6:**
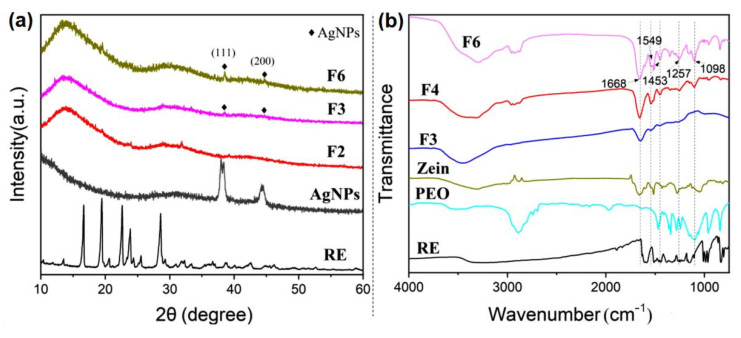
Physical status and compatibility of antibacterial nanofiber membranes: (**a**) XRD analysis; (**b**) FT-IR spectra.

The physical morphology of the fiber and raw materials was characterized by XRD diffraction, as shown in Figure 6a. F3 represented the uniaxial fiber loaded with nanosilver, and F6 represented the coaxial fiber loaded with nanosilver and resveratrol simultaneously. It was found that the RE raw material had multiple sharp peaks, indicating its crystalline structure. However, these crystalline characteristics were not found in the RE-loaded fiber, which showed that the physical state of RE existed in the fiber changed from a crystalline state to amorphous state. For nanosilver, when the angle of 2θ was about 38° and 44°, the characteristic peaks appeared. They corresponded to the (111) and (200) crystal planes of silver, respectively. Additionally, these two above-characteristic peaks of silver were also found in the fibers loaded with silver nanoparticles, proving the successful loading of silver nanoparticles. In the fiber, due to the low content of silver nanoparticles, the peak intensity was weak, and the peak of F3 was even weaker than that of F6. It was inferred that the loading capacity of uniaxial zein was poor, which might be mainly due to the irregular morphology of uniaxial fiber, whereas the coaxial fiber possessed a more uniform and stable morphology that was beneficial for the good dispersion of nanoparticles in the fiber.

The compatibility of the internal components of the fiber was analyzed by Fourier transform infrared reflection. F3, F4 and F6 were analyzed for the comparison of the uniaxial fiber loaded with AgNPs, coaxial fiber loaded with AgNPs and coaxial fiber loaded with AgNPs and RE. Obvious characteristic peaks at 1668, 1549, 1453, 1257 and 1098 cm^−1^ were mainly found in the F6 fiber, which represented the vibration of the -C=O, C=N, -CH_3_, -C-O and C-OH functional groups, respectively. Meanwhile, the high peaks were also found in the F5 fiber in which resveratrol was not loaded. Through comparison, it could be analyzed that the carbonyl peak at 1668 cm^−1^ and the amino peak at 1549 cm^−1^ came from the zein protein. The methyl and carbonyl groups represented at 1453, 1257 and 1098 cm^−1^ mainly came from PEO, which determined that PEO and zein were the matrix materials of fiber. After adding resveratrol to F3, there were no more characteristic peaks of RE at 1000–1700 cm^−1^ in the F5 fiber, indicating that there was chemical bonding that occurred between RE and the matrix with good compatibility. Furthermore, it could be found that the methyl and carbonyl peaks at 1453 and 1257 cm^−1^ in the fiber shifted from the wavenumber of the original position that occurred in raw-material PEO and the amino peak at 1549 cm^−1^ shifted from the wavenumber compared to that from zein. The phenomenon indicated that the vibration frequency of functional groups had changed. Therefore, it could be inferred that there was a hydrogen bond between PEO, zein and RE.

**Figure 7:**
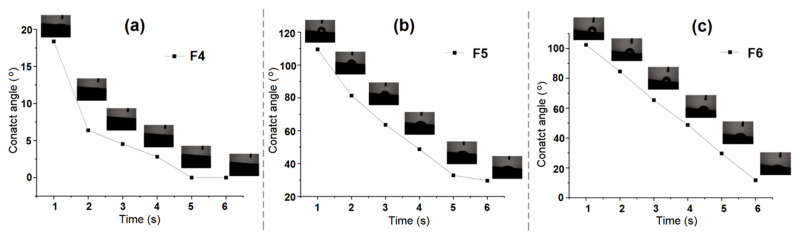
Water contact angle of nanofiber membranes: (**a**) F4; (**b**) F5; (**c**) F6.

In the static water contact angle analysis, as shown in Figure 7, the water contact angle of the F4 fiber was 18.4° within 1 s of contacting, and the water droplets were completely spread on the fiber at 5 s. Therefore, the F4 fiber had strong hydrophilicity, which revealed that the coaxial electrospun fiber possessed strong water permeability. Although zein is generally hydrophobic, there were some hydrophilic amino acids in the protein structure [78]. These amino acids allowed water molecules to penetrate into the fiber to reach the fiber core layer, namely, the hydrophilic PEO layer, which enabled the fiber to be more hydrophilic as a whole. In F5 and F6, after resveratrol was added to the fiber core layer and fiber sheath layer in turn, the fiber hydrophobicity was enhanced. For the existence of the water contact angle, F5 decreased from 109.5° to 29.6° during the time points from 1 s to 6 s, and F6 decreased from 102.4° to 11.7° at the same time. It could be considered that the added hydrophobic active substance, resveratrol, enhanced the overall hydrophobicity of the fiber. Moreover, it was observed from the slope of the curve that the water contact angle of F6 was verified to be more even with time, which indicated that the fiber delayed the water absorption from the surface to the inner layer in the whole fiber structure, rather than delaying the water absorption of F5 from fast to slow. In the process of packaging fruit, the fruit would consume its own organic substance through certain respiration and release carbon dioxide and water inevitably [79,80]. As a result, more or less condensed water would be generated in the closed packaging environment after a certain time, which could be undesirably helpful to the reproduction of microorganisms [81]. Therefore, the F6 fiber with a certain moisture absorption could effectively eliminate these water droplets to prevent bacterial reproduction and further promote the dissolution and diffusion of resveratrol molecules in the packaging environment.

### 3.4. Surface Water Contact Property of Functional Ultrathin Fibers

**Figure 8:**
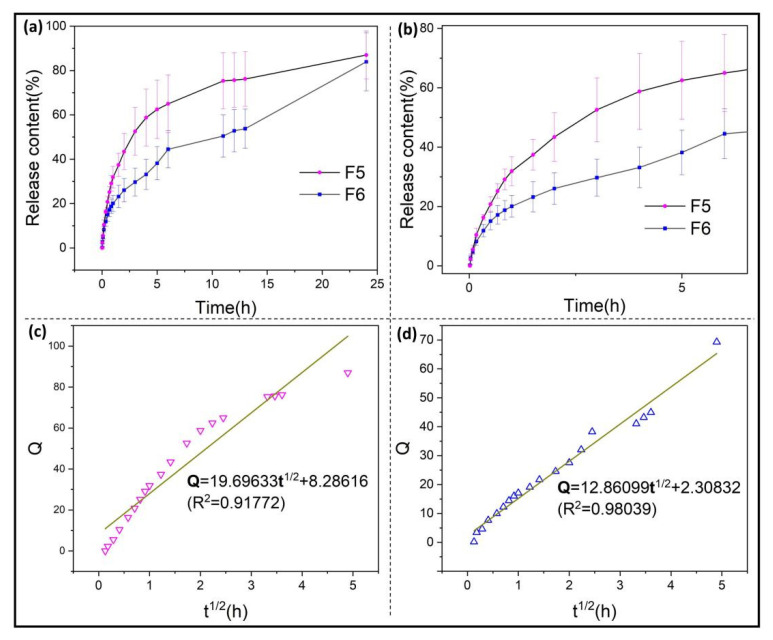
Release profiles of electrospun nanofiber membranes: (**a**) release characteristics of F5 (coaxial fiber loaded with RE in the core structure) and F6 (coaxial fiber loaded with RE in both core and sheath structure) in 24 h; (**b**) partial enlarged view of F5 and F6 curve in 6 h; (**c**) regressed equation of RE release profile from F5; (**d**) regressed equation of RE release profile from F6.

The dissolution content of RE from fiber contact in deionized water was measured to determine the release characteristics of RE in the packaging environment from water absorption. As shown in Figure 8a, the final release content of RE from F5 and F6 was 86.99 ± 10.80% and 83.94 ± 13.10%, with both reaching a relatively sufficient release. Among them, the F5 fiber had a fast release rate, which indicated the water permeability of the fiber sheath structure. Water molecules entered the fiber to dissolve the PEO core layer and promoted the release of RE molecules. The F6 fiber with RE added to both core and sheath layers presented the characteristics of slow release. It could be inferred that, based on the RE release characteristics of F5, the release rate of RE molecules from F6 in the zein sheath was slow and RE was well encapsulated in the fiber sheath. After being contacted by water molecules, it could remain stable without escaping from the fiber; thus, the fiber could release enough RE on average within 24 h to achieve a continuous, efficient packaging effect. As shown in Figure 8b, the RE content released with half an hour from F5 and F6 was 20.79 ± 2.40% and 15.07 ± 2.99%, respectively; with there being a small difference, this indicates that the release of RE from F5 and F6 was basically dominated by the dissolution release of core PEO before 0.5 h. On the other hand, F5 and F6 released 64.98 ± 13.01% and 44.52 ± 8.37%, respectively, within 6 h. Compared with the final release, F5 had released about three quarters, whereas F6 released about half, showing that F6 had a strong persistent release ability. As shown in Figure 8c,d, the dissolution curves of F5 and F6 were fitted by linear regression through the Higuchi model function. The closer the fitting points were to the linear distribution, the better they could be explained to be closer to the sustained release [82,83,84]. The regression curves of F5 and F6 were Q = 19.69633 t^1/2^ + 8.28616 (R^2^ = 0.91772) and Q = 12.86099 t^1/2^ + 2.30832 (R^2^ = 0.98039). F6 reached the sustained-release characteristics closer to the standard. Therefore, it could be speculated that under a certain humidity when actually packaging food, the RE release time of F5 and F6 would be amplified multiple times; therefore, F6 could enable more effective packaging in the time of several more days than F5, which is further confirmed in the following experiments.

### 3.5. Release Behavior of Resveratrol from Functional Ultrathin Fibers

**Figure 9:**
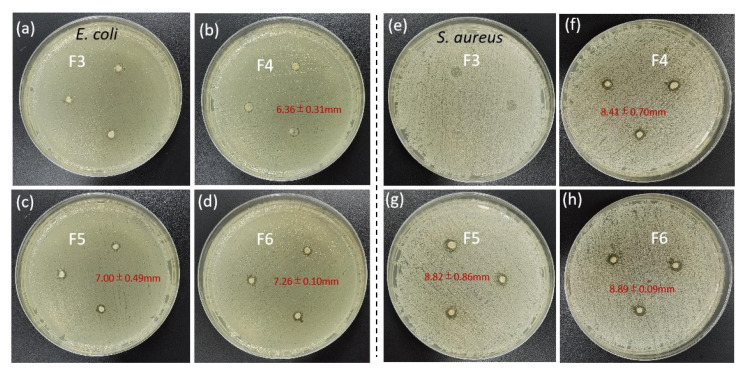
Inhibition zones of electrospun nanofiber membranes: (**a**–**d**) antibacterial effect of F3–F6 on *E. coli*; (**e**–**h**) antibacterial effect of F3–F6 on *S. aureus*.

*Staphylococcus aureus* and *Escherichia coli* were used as test bacteria to evaluate the antibacterial activity of F3–F6. As shown in Figure 9, when observing the bacteriostatic effect on *E. coli*, it was observed that the antibacterial activity of F3 was not obvious, and there were some punctuated, discontinuous bacteriostatic traces around the disc, which might be due to the insufficient load of nanosilver caused by the unstable string structure of the uniaxial zein fiber. The F4 coaxial fiber showed an obvious inhibition zone with a diameter of 6.36 ± 0.31 mm, indicating that the nanosilver was more well and evenly dispersed on the surface of the nanofiber so that the bacteria contacted around the disc could be killed. In the observation of F5 and F6, it was found that the area of the inhibition zone increased with a diameter of 7.00 ± 0.49 mm and 7.26 ± 0.10 mm, which showed that the antibacterial activity of the fiber was increased with the addition of RE, and the RE molecules were distributed around the fiber disc through diffusion and prevented the bacteria from approaching. In the comparison of F5 and F6, it was found that the increase in RE content further increased the diameter of the inhibition zone. Similarly, in the inhibition test of Staphylococcus aureus, the above conclusions could also be drawn, with an inhibition zone diameter of 8.41 ± 0.70 mm, 8.82 ± 0.86 mm and 8.89 ± 0.09 mm. It could be concluded that the contacting antibacterial effect of nanosilver on the fiber surface was effectively combined with the molecular diffusion antibacterial effect of resveratrol, resulting in excellent antibacterial activity of the fiber film. In the comparison of the two bacteria, the inhibitory zone of fiber on Staphylococcus aureus was more clear, which revealed that nanosilver and resveratrol possessed a stronger inhibitory effect on Staphylococcus aureus. Moreover, because of the fact that Staphylococcus aureus was from a foodborne pathogenic microorganism [85], it is desirable for there to be good antibacterial activity against it as that is what is properly needed for food packaging. Compared with other studies, it was found that the inhibition zone in this experiment was not too large, which was caused by the relatively small disc mass; thus the active substance might not be enough to play a role. Despite the small amount of antibacterial substance, the regularity was still discovered in the inhibition zone test, reflecting the uniformity of active substances loaded in the fiber membrane from the side. Antibacterial materials are highly desired in many applied fields [86,87,88], and the combinations of inorganic nanoparticles (such as AgNPs) and small active molecules (such as RE) often result in a synergistic effect. Based on this strategy and the reasonable applications of AgNPs [89,90,91], many new food packaging membranes will be possible in the future.

### 3.6. Antibacterial Activity of Functional Nanofibers

**Figure 10:**
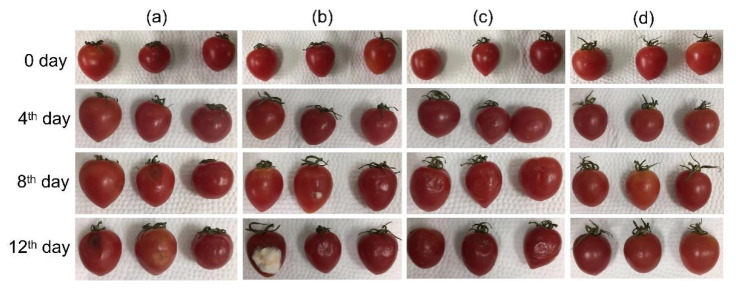
Package application of cherry tomatoes: (**a**) controlled group without packaging; (**b**) F4-packaged group; (**c**) F5-packaged group; (**d**) F6-packaged group.

In order to evaluate the effect of fiber film in practical packaging, cherry tomatoes were selected as the packaging object. The packaging of cherry tomatoes with fiber film within 12 days was studied at room temperature (25 ± 1 °C). As a perishable fruit, cherry tomatoes are worthy of antibacterial packaging, which is conducive to prolonging the shelf life of cherry tomatoes [92]. As shown in Figure 10, one cherry tomato in the blank group showed black spots from the 8th day, whereas one cherry tomato in the F4 packaging group grew white mold, which even increased by the 12th day. It could be caused by a bacterial infection after a tiny rupture in the tomato epidermis, indicating that the antibacterial performance of the F4 fiber loaded with nanosilver was not enough. It was further found that a cherry tomato packed with F5 fiber appeared to have black spots on the 12th day, but no bacterial infection was found in the F6 fiber packaging, revealing that the antibacterial activity of the fiber gradually increased with the increase in RE content. From the fourth day, the cherry tomatoes in the first three groups appeared to have surface wrinkles in varying degrees. It was considered that microorganisms invaded the interior of the fruit from the slight cracks on the peel, resulting in the decomposition of nutrients [93]. The surface of the cherry tomatoes packed with F6 fiber was flat and smooth until the 12th day, reflecting the excellent antibacterial performance of the F6 fiber to keep cherry tomatoes fresh.

### 3.7. Practical Application of Packaging Cherry Tomatoes

**Figure 11:**
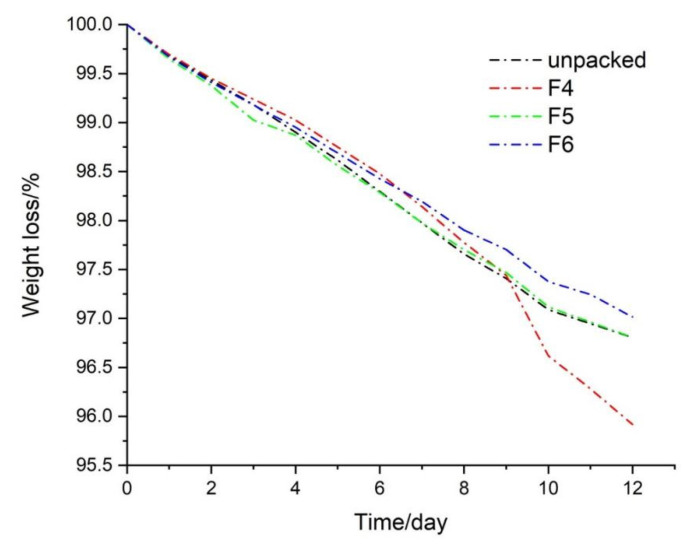
Weight loss of cherry tomatoes in the package application.

The weight loss curve of the cherry tomatoes was measured to test the freshness of the cherry tomatoes. As shown in Figure 11, on the 12th day, the weight loss rate of the cherry tomatoes packaged in F4 was the highest, decreased to 95.92% from the original quality. The reason was too much mold growth from one parallel sample in this group; thus, the nutrients in the cherry tomatoes were largely decomposed, resulting in the low quantity of the data. On the contrary, the weight loss rate of the cherry tomatoes packaged with the F6 fiber was the lowest, down to 97.02%, which showed that the loss of nutrients and water of cherry tomatoes packaged with the F6 fiber was the smallest, and they were kept fresh effectively. The quality of cherry tomatoes in the blank group and F5 packaging decreased to 96.805% and 96.807%, respectively. Both weight loss rates were almost the same. Therefore, it could be considered that under the closed environment, the ethylene released by the F5 fiber packaging the cherry tomatoes accumulated in the environment, and kept on accelerating its own respiration, which meant the accelerated consumption of water and nutrients [94]. Although the ethylene released by the cherry tomatoes in the blank group could circulate into the air, the erosion by more microorganisms of the cherry tomatoes could not be resisted without the antibacterial package, resulting in the decline of quality. In summary, despite the issue of ethylene release in the package, the relative excellent antibacterial ability of F5 was reflected.

After 12 days of the cherry tomatoes being packaged, the total number of bacterial colonies on the surface of the cherry tomatoes was measured to qualitatively determine the antibacterial effect of the fiber membrane on cherry tomatoes. As shown in Table 2, Sample 1 of F4 had a relatively high total number of colonies, which corresponded to the individual cherry tomato with white mold growth. The rupture of the individual’s epidermis led to the proliferation and reproduction of bacteria that, depending on the nutrients flowing out from it, acted as the culture medium. Therefore, after excluding this error value, the total number of bacterial colonies of cherry tomatoes packaged in F4 decreased from 5.9555 to 2.3542 CFU/g, which was smaller than the total number of colonies in the blank group and larger than that of F5-packaged cherry tomatoes, which was to be expected. It can be concluded that with the increase in the content of antibacterial active substances, the antibacterial activity of the fiber membrane packaging increased correspondingly, indicating that nanosilver and resveratrol had a synergistic effect on their antibacterial activity. Along this proof-of-concept demonstration and with the capability of electrospinning in creating complex nanostructures such as Janus particles, a tri-layer core-sheath and their combinations [95,96,97], many novel fiber membranes loaded with multiple active ingredients will be further developed for food packaging in the future, particularly from many bioactive molecules [98,99].

## 4. Conclusions

In this paper, a novel zein/PEO ultrathin fiber was successfully prepared by coaxial electrospinning technology. SEM and TEM showed that the fiber had a clear core-sheath structure, smooth surface and uniform diameter distribution. In addition, through component analysis, the active substances of nanosilver and resveratrol were proven to be in the fiber. Nanosilver existed in the fiber sheath in the form of embedding, and hydrogen bonds were generated between resveratrol molecules and polymers along with good compatibility. The water contact angle of the fiber decreased from 102.4° to 11.7° in 6 s, indicating that the fiber possessed stable moisture absorption. For the dissolution experiment in vitro, the release characteristics of resveratrol showed a diffusion mechanism within 24 h. The antibacterial activity against *S. aureus* and *E. coli* was effective, which was 7.26 ± 0.10 mm and 8.89 ± 0.09 mm, respectively. In the experiment of packaging cherry tomatoes, the good antibacterial performance of the fiber was further verified. Under the interactive antibacterial effect of resveratrol and nanosilver, the fiber membrane effectively inhibited the growth of bacteria on the surface, reduced the loss of quality and prolonged the freshness preservation period of cherry tomatoes within 12 days. It could be used as an effective food antibacterial packaging material.

## Figures and Tables

**Table 1:** Electrospinning processing parameters for prepared fibers.

Fiber No.	Electro- Spinning ^a^	Applied Voltage (kV)	Core Fluid ^b^			Sheath Fluid ^c^			Product
			Polymer	Functional Ingredients	Flow Rate (mL/h)	Polymer	Functional Ingredients	Flow Rate (mL/h)	
F1	Single-fluid	5.5	PEO	-	0.8	-	-	-	Fibers
F2	Coaxial	6.0	PEO	-	0.6	Zein	-	0.3	Fibers
F3	Single-fluid	17	-	-	-	Zein	AgNPs	0.2	Beads-on-a-string
F4	Coaxial	7.0	PEO	-	0.6	Zein	AgNPs	0.3	Fibers
F5		7.0	PEO	RE	0.6	Zein	AgNPs	0.3	
F6		7.0	PEO	RE	0.6	Zein	AgNPs + RE	0.3	

^a^ The collection distance of all the electrospinning processes is set at 23 ± 2 cm. ^b^ The concentration of PEO in the core fluid is 3% (*w*/*v*), with RE at 10% (*w*/*w*) relative to the polymer. The solvent is the mixture of ethanol and deionized water (80:20 in volume). ^c^ The concentration of zein in the sheath fluid is 28% (*w*/*v*), with AgNPs at 0.1% (*w*/*w*) and RE at 5% (*w*/*w*) relative to the polymer. The solvent is the mixture of ethanol and deionized water (80:20 in volume).

**Table 2:** Bacterial test parameters of cherry tomatoes after 12th packaging.

Sample	Parallel Group Number	Bacterial Content (CFU × 10^6^)	Sample Mass (g)	Bacterial Content per Mass (CFU/g × 10^5^)
Unpacked	1-1	2.2	11.32	1.9435
	1-2	2.6	8.09	3.2138
	1-3	3.4	7.92	4.2929
	Mean value			3.1501 ± 1.1760
F4	2-1	15	11.40	13.1579
	2-2	3.5	10.12	3.4585
	2-3	1.3	10.40	1.2500
	Mean value			5.9555 ± 6.3345
F5	3-1	0.4	8.46	0.4728
	3-2	2.0	6.98	2.8653
	3-3	1.8	9.61	1.8730
	Mean value			1.7371 ± 1.2020
F6	4-1	1.8	9.46	1.9027
	4-2	1.6	10.13	1.5795
	4-3	1.3	9.97	1.3039
	Mean value			1.5954 ± 0.2997

## Data Availability

No applicable.
